# Multivariate dynamical modelling of structural change during development

**DOI:** 10.1016/j.neuroimage.2016.12.017

**Published:** 2017-02-15

**Authors:** Gabriel Ziegler, Gerard R. Ridgway, Sarah-Jayne Blakemore, John Ashburner, Will Penny

**Affiliations:** a Institute of Cognitive Neurology and Dementia Research, Otto-von-Guericke-University Magdeburg, 39120 Magdeburg, Germany; b German Center for Neurodegenerative Diseases (DZNE), 39120 Magdeburg, Germany; c FMRIB Centre, University of Oxford, Nuffield Department of Clinical Neurosciences, John Radcliffe Hospital, Headington, Oxford OX3 9DU, UK; d Institute of Cognitive Neuroscience, University College, London WC1N 3BG, UK; e Wellcome Trust Centre for Neuroimaging, University College, London WC1N 3BG, UK

**Keywords:** Bayesian inference, Dynamical systems, Longitudinal analysis, Structural brain mapping, Brain maturation, Ageing, Connectivity

## Abstract

Here we introduce a multivariate framework for characterising longitudinal changes in structural MRI using dynamical systems. The general approach enables modelling changes of states in multiple imaging biomarkers typically observed during brain development, plasticity, ageing and degeneration, e.g. regional gray matter volume of multiple regions of interest (ROIs). Structural brain states follow intrinsic dynamics according to a linear system with additional inputs accounting for potential driving forces of brain development. In particular, the inputs to the system are specified to account for known or latent developmental growth/decline factors, e.g. due to effects of growth hormones, puberty, or sudden behavioural changes etc. Because effects of developmental factors might be region-specific, the sensitivity of each ROI to contributions of each factor is explicitly modelled. In addition to the external effects of developmental factors on regional change, the framework enables modelling and inference about directed (potentially reciprocal) interactions between brain regions, due to competition for space, or structural connectivity, and suchlike. This approach accounts for repeated measures in typical MRI studies of development and aging. Model inversion and posterior distributions are obtained using earlier established variational methods enabling Bayesian evidence-based comparisons between various models of structural change. Using this approach we demonstrate dynamic cortical changes during brain maturation between 6 and 22 years of age using a large openly available longitudinal paediatric dataset with 637 scans from 289 individuals. In particular, we model volumetric changes in 26 bilateral ROIs, which cover large portions of cortical and subcortical gray matter. We account for (1) puberty-related effects on gray matter regions; (2) effects of an early transient growth process with additional time-lag parameter; (3) sexual dimorphism by modelling parameter differences between boys and girls. There is evidence that the regional pattern of sensitivity to dynamic hidden growth factors in late childhood is similar across genders and shows a consistent anterior-posterior gradient with strongest impact to prefrontal cortex (PFC) brain changes. Finally, we demonstrate the potential of the framework to explore the coupling of structural changes across *a priori* defined subnetworks using an example of previously established resting state functional connectivity.

## Highlights

• Multivariate approach for structural changes in development using dynamical systems.
• States follow a system with inputs accounting for driving forces of development.
• Demonstrate dynamic gray matter changes during maturation between 6 and 22 years.
• Account for effects of puberty and estimate a hidden growth factor affecting PFC.
• Explore coupled structural change within/between two resting-state fMRI networks.

## 1 Introduction

The human brain undergoes profound structural changes during development and aging. Magnetic resonance imaging (MRI) has become an invaluable tool to measure these brain changes *in vivo*. There is an increasing number of advanced longitudinal neuroimaging projects that focus on the specific patterns of change during brain maturation and development (for review see Mills and Tamnes, 2014). Several aspects of brain anatomy have been reported to undergo curvilinear changes with different markers progressing differently during development (Giedd et al., 1999, Lenroot et al., 2007, Raznahan et al., 2011, Mills et al., 2016). Recent studies indicate that cortical gray matter volume exhibits its highest volume during mid-to-late childhood, and decreases across the second decade (Tamnes et al., 2013, Aubert-Broche et al., 2013, Wierenga et al., 2014, Mills et al., 2016). There is also longitudinal evidence for gender differences in the shapes of developmental trajectories, with peak sizes 1 to 2 years earlier in females (Lenroot et al., 2007), although these differences are reduced when overall cranial volume is taken into account in the statistical model (Mills et al., 2016). Converging findings from cross-sectional and longitudinal studies in late childhood and adolescence also suggest that puberty-related physiological and hormonal changes induce brain changes in specific networks (Blakemore et al., 2010).

The primary goal of the current study was to develop a novel modelling framework rather than clarifying phenotype-specific questions about brain trajectories of regional gray matter volumes. One limitation of most previous studies on structural development is that mass-univariate techniques like general linear models (GLM) or linear-mixed models (LME) (Bernal-Rusiel et al., 2012, Ziegler et al., 2015) are applied. That often involves whole brain explorative analysis in order to identify local structural correlates of age or time, which survive a correction for multiple comparisons. The analysis of region-specific effects is often followed by a *post hoc* discussion and integration of observed results across multiple brain regions, which involves potential anatomical, physiological and neurological causal factors. In this context, terms such as ‘states’, ‘processes’ and ‘trajectories’ are used rather informally in the literature.

Here we introduce the characterisation of structural imaging data using multivariate differential equation models. This general approach will allow us to study the structural changes underlying brain development, plasticity, ageing and degeneration from a dynamical systems perspective. In our approach ‘states’ and ‘trajectories’ then take on a precise meaning endowed by the formal specification of a dynamical system with input factors. Our framework avoids serious limitations of univariate models, e.g. multiple testing, by providing a multivariate model for a whole set of brain regions under Bayesian inference. With regard to structural dynamics, states, *x*, would correspond to a vector of structural indices (e.g. gray matter volumes) in a set of brain regions at a single time point. The system then responds to inputs, *u*, a vector of values at a single time point comprising for example levels of hormones, growth factors or proteins. The change in state is then given by

(1) $$\frac{\mathrm{dx}}{\mathrm{dt}}=f(x,u,a)$$

where f(x,u,a) describes a dynamical process governed by parameters *a*. These parameters define, for example, the time constants of interactions among states. Most generally the states may only be observable through a noisy observation function y=g(x,w)+e. This overall description corresponds to the multiple-input-multiple-output (MIMO) system described previously (Friston et al., 2003, Friston, 2002).

In the study of development, hormonal or growth factor variables *u* would perturb states leading to periods of maximal growth. Such models are readily able, for example, to describe the logistic, multiple-logistic and other patterns of growth observed in biology (Thompson and Growth, 1945, Murray, 2002). Additionally, interactions among state variables might account for regional patterns of volumetric change arising from synaptic growth and pruning. This would add value to current univariate perspectives on structural changes (reviewed in Mills and Tamnes 2014), by adding a multivariate and dynamic perspective. It also relates to the recently proposed notion of ‘maturational coupling’, i.e. exploring similarities of changes across brain regions (Raznahan et al., 2011), but in principle should additionally enable quantification of joint underlying processes. Our proposal shares the ambitions of the Dynamical Bayesian Network (DBN) approach for studying inter-regional dependencies in structural brain imaging (Chen et al., 2012). The DBN approach operates in discrete time and models discrete observations (e.g. stable/atrophy), whereas the MIMO approach operates in continuous time and models continuous observations (e.g. gray matter volumes).

The DBN and MIMO frameworks share the benefits of a nonlinear dynamical systems perspective, thus going beyond linear-mixed effects models. However, being based on differential equations, the MIMO approach is closer to standard approaches in systems biology and neuroscience (Dayan and Abbott, 2001, Deco et al., 2008, Ingalls, 2013). Indeed, the work in this paper uses the same model estimation and inference algorithm (‘Variational Laplace’ (Friston et al., 2007)) that is incorporated in the Dynamic Causal Modelling (DCM) framework for making inferences about changes in brain connectivity from fMRI (Friston et al., 2003) or M/EEG data (Daunizeau et al., 2009).

In what follows, we describe sample specifics, details about longitudinal MR image processing, and the specification of system inputs. Then we introduce the specifics of the proposed model, briefly revisiting the procedures for inference. In the later sections of the paper we aim to demonstrate the construct validity of a dynamical systems approach in the context of brain maturation using a large sample of healthy children and adolescents. We present model estimates and examples for evidence-based model comparison using the empirical data. We hypothesise that intrinsic regional dynamics in development can be described using a multivariate linear dynamical system. According to previous findings we also expect substantial contributions of a puberty-related factor and a growth factor to the regional gray matter dynamics. Finally, using our novel approach we study an example of inter-regional connectivity and whether structural changes during development do reflect functional networks previously observed in resting state fMRI (Smith et al., 2009).

## 2 Methods

### 2.1 Sample

For the purpose of validation with real data, we used a subsample of the NIH Pediatric MRI Data Repository created by the NIH MRI Study of Normal Brain Development (Evans and Group, 2006). This project focuses on brain development in healthy typically developing infants, children and adolescents from a demographically balanced population based sampling. The data was acquired in multiple pediatric centers and included a variety of MR-based sequences and protocols (https://nihpd.crbs.ucsd.edu). A major part of the project aims at exploring the general course of normal brain development. Notably, the screening procedures excluded subjects with a family history of inherited neurological disorders or a lifetime history of Axis I psychiatric disorders, abnormalities during perinatal development, birth complications, physical growth problems, neurological or specific psychiatric disorders. A detailed description of the full sample acquisition and exclusion criteria can be found in Evans and Group (2006).

Image processing started with a sample from release 5 of the NIH MRI study objective 1 of the children and adolescents. The sample downloaded from the NIH repository included 770 scans of 401 subjects scanned at ages 4.8–21.9 years with zero, one or two annual follow-up scans per subject. A detailed overview of the acquisition protocols of the NIH MRI Study of Normal Brain Development can be found here (http://pediatricmri.nih.gov/nihpd/info/protocols.html) and in Evans and Group (2006). The available sample included data from both primary protocols and fallback protocols with either 1 mm or 3 mm slice thickness, respectively. We observed variations in raw data slice resolution influencing the quality of the image preprocessing results and discarded further 32 scans due to any serious artifacts in image segmentation, registration, or nonlinear normalization. After MR preprocessing we quality checked the image data (for details see Section 2.3). Indications for lower data quality, higher frequency of usage of the fallback (rather than the standard) protocols, and much sparser density of sampling at the lower age range resulted in discarding children younger than six years. We further focussed on a longitudinal sample for validation of our dynamical systems model, i.e. we included only subjects having follow-up measurements. The analyzed sample consisted of 289 children and adolescents (151 females, 135 males) with ages 6–21.9 years (M=12.47, SD=3.88 years) with in total 637 scans (338 from females and 299 obtained from males), 2–3 scans per subject.

### 2.2 Pubertal status

As a proof of principle we included a puberty-related input factor to the dynamical model of brain maturation. The NIH Pediatric Repository provides access to pubertal status questionnaire scores. This method is similar to the Tanner staging that is widely used for assessing physical puberty stage of individuals (Taylor et al., 2001). Here, girls (and boys) were rated with respect to the current expression of physical pubertal features, in particular, height growth spurt, body hair growth, skin changes, breast growth (voice deepening), menstruation start (facial hair) respectively. A score was assigned on the stage that the participant felt best described themselves using (1=Not Started; 2=Barely Started; 3=Definitely Under Way; 4=Completed). A summary score was calculated using the mean expression across all 5 features of expression for each individual. After checking for completeness, the puberty status summary score was available for 411 subjects (214 females, 197 males) of the NIH pediatric sample at 889 acquisition timepoints (476 females, 413 males). The final summary scores were rescaled to a [0,1] interval with higher values indicating higher pubertal stage. In order to define a group level quantitative model of pubertal transition from no signs of puberty to full expression of external primary and secondary gender characteristics, we fit sigmoid curve models using nlinfit to the rescaled observations (MATLAB R2014b, MathWorks, http://uk.mathworks.com/products/matlab/index.html). In particular, the following sigmoid model was fit to both gender groups independently

(2) $$h(t,p)=\frac{1}{1+10^{(p_{1}-t)p_{2}}}$$

with age (or time) variable *t*, age of strongest change *p*_1_ and slope *p*_2_ as free parameters. In what follows the temporal derivative of the obtained sigmoid pubertal transition function *h* is considered as a proxy for puberty-related physiological hormones and growth factors. Therefore we here use *h* to define the actual forces causing puberty-related changes in our dynamic brain model by

(3) $$u_{1}(t)=\frac{\mathrm{dh}}{\mathrm{dt}}.$$

To create the puberty inputs for boys and girls for the dynamical model the parameters *p*_1_ and *p*_2_ were set to the gender-group specific estimates described above.

### 2.3 Longitudinal image processing

All further preprocessing steps were performed in SPM12 r6685 (Wellcome Trust Centre for Neuroimaging, London, UK, http://www.fil.ion.ucl.ac.uk/spm). Longitudinal MR-based morphometry is particularly prone to artifacts due to scanner inhomogeneities, registration inconsistency, and subtle age-related deformations of the brains. Therefore, we used a specifically designed longitudinal preprocessing pipeline in order to detect the changes of interest and achieve unbiased results during the subsequent dynamical systems modelling.

Firstly, we applied the symmetric diffeomorphic registration of serial MRI (Ashburner and Ridgway, 2013). The registration model creates a midpoint T1-image for each subject and the corresponding deformation fields from this midpoint to each acquired scan at all timepoints.

Second, we used SPM12's unified segmentation of the individual midpoint images, classifying the T1 into gray matter (GM), white matter (WM), and cerebrospinal fluid (CSF), bone, other tissue and air classes (Ashburner and Friston, 2005).

Third, all segmented tissue maps in midpoint space were multiplied by the Jacobian determinants from the within-subject deformation fields in order to account for volume changes over time. This step is often called Jacobian modulation and is used here to create a set of aligned segmentations whose volumes reflect those of the original time-points, but with reduced error variability due to the use of a single segmentation step on the midpoint average images that have higher signal-to-noise ratio and greater anatomical precision compared to the individual time-point images.

Fourth, nonlinear template generation and image registration of the individual midpoint images was performed using the DARTEL algorithm (Ashburner, 2007).

Fifth, in order to avoid introducing systematic bias due to errors in any of the above steps we further quality checked the processed data using covariance-based inhomogeneity measures of the sample as implemented in the CAT12 r937 SPM toolbox (Structural Brain Mapping Group, Departments of Psychiatry and Neurology, Jena University Hospital,http://dbm.neuro.uni-jena.de/cat/).

Sixth, since we intended a dynamical model of large portions of the regional gray matter tissue, we focussed on an atlas providing a high quality parcellation of this tissue class including cortical and subcortical regions. Notably, in context of this first approach to multivariate dynamical models during development, we restricted the total number of regions to the order of tens rather than hundreds. A reasonable compromise between whole brain coverage and number of regions is provided by the Hammer's probabilistic brain atlas (Hammers et al., 2003) and restricting the analysis to bilateral ROIs assuming similar development in homologous brain regions. We registered this atlas with the generated template and deformed it into the individual midpoint spaces (obtained from longitudinal registration). Voxelwise (within-subject modulated) gray matter segments in the midpoint space were summed up within 26 bilateral ROIs: Insula (Ins), anterior cingulate gyrus (AntCinG), posterior cingulate gyrus (PosCinG), frontal gyrus (FroG), inferior frontal gyrus (InfFroG), middle frontal gyrus (MidFroG), superior frontal gyrus (SupFroG), precentral gyrus (PrcG), rectal gyrus (RecG), anterior medial temporal lobe (AntMedTemL), anterior lateral temporal lobe (AntLatTemL), superior temporal gyrus (SupTemG), inferior middle temporal gyrus (InfMidTemG), fusiform gyrus (FusG), posterior temporal lobe (PosTemL), post central gyrus (PoCG), lateral parietal lobe (LatParL), superior parietal gyrus (SupParG), cuneus (Cun), lateral occipital lobe (LatOccL), lingual gyrus (LinG), hippocampus (Hip), amygdala (Amy), putamen (Put), caudate nucleus (CauNuc), and thalamus (Tha). This steps determines the absolute volumes of each ROI including variability due to potential volume changes within- and between subject.

Finally, in a separate step the above whole brain segmentations were used to approximate total intra-cranial volume (TICV) as a sum of GM, WM and CSF tissue classes. Since neuroanatomic correlates might be influenced by confounds e.g. global brain parameters (Taki et al., 2012, Peelle et al., 2012), TICV was used as a confounding variable during model inversion accounting for brain size differences across subjects and age. The regional volumes were further rescaled to [0,100] to simplify comparisons of effects of time/age across ROIs with different sizes. All subsequent dynamical modeling steps were performed on 26 bilateral ROI volumes obtained from using the above steps.

### 2.4 Dynamical model

In what follows N(m,Λ) denotes a multivariate Gaussian variable *x* with mean *m* and precision *Λ*. Readers unfamiliar with terminology from dynamical systems are referred to standard texts (Wilson, 1999).

**Fig. 1:**
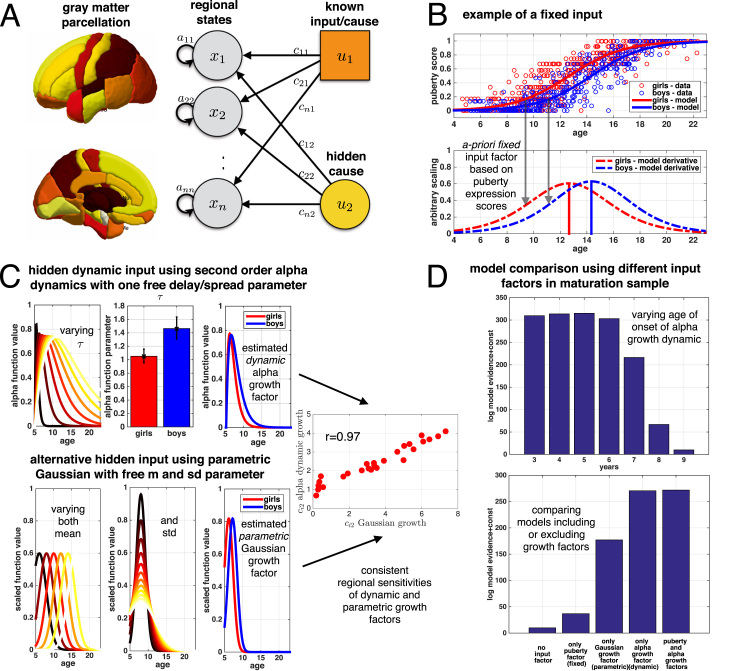
Model overview and growth factors considered to drive the structural changes. A An illustration of the model of structural changes and its two distinct inputs. B Input factor using puberty-related sigmoidal curves $h(t,p_{1},p_{2})$ (see Eq. (2)) to rescaled puberty-related summary scores in 214 girls (red) and 197 boys (blue) (top plot in B). The actual pubertal input factor was assumed to be the temporal derivative of the sigmoidal curves, i.e. $u_{1}=\mathrm{dh}/\mathrm{dt}$ (bottom plot in B). Highlighted are the ages of fastest change of pubertal signs, *p*_1_, with 12.66 years for girls and 14.34 years for boys. C Top row shows the hidden growth factor implemented using alpha function dynamics with one free delay parameter *τ*. Brighter functions indicate delayed and more enduring growth (top left). Obtained posterior of parameter *τ* using 289 subjects (top middle). Estimated shape of alpha growth factor shown for girls (red) and boys (blue) (top right). C Bottom row illustrates an alternative parametric implementation of a hidden growth factor using a Gaussian with one free mean for each gender (bottom left) and one additional free standard deviation parameter (bottom middle). Estimated shape of Gaussian growth factor shown for girls (red) and boys (blue) (bottom right) using 289 subjects. Regional sensitivities *c*_*i2*_ obtained from dynamic and parametric growth factors. D Bayesian model comparison of alpha growth with different age of onset (top) and comparing alternative inputs to the dynamical model (bottom).

In general, the evolution (or change) of structural states in region *i* is modelled as a linear function of the current states of connected regions (usually including a self-connection) and external inputs, i.e.

(4) $$\frac{{\mathrm{dx}}_{i}}{\mathrm{dt}}=\overset{D}{\underset{k=1}{\sum }}a_{\mathrm{ik}}x_{k}+\overset{J}{\underset{j=1}{\sum }}c_{\mathrm{ij}}u_{j}$$

where *x*_*k*_ is e.g. gray matter volume in region *k*, *a*_*ik*_ are self- and between-region connection parameters, *u*_*j*_ is the *j*th input variable, and *c*_*ij*_ the regional sensitivity of region *i* to the *j*th input. The connectivity matrix *A* with entries *a*_*ik*_ then allows flexible implementation of models with uncoupled intrinsic dynamics (diagonal entries only, *a*_*ii*_) or more complex models with interactions among regions. For models of development we envisage that inputs *u*_*j*_ correspond to various neurotrophic growth factors and that the regional sensitivity parameters *c*_*ij*_ relate to the regional density of receptors of region *i* for growth factor *j*, or otherwise indicate region-specific molecular and biochemical susceptibility to the factor's effects. See Discussion for further elaboration. An illustration of the model and the considered inputs can be seen in Fig. 1A, B and C.

In the following validation in the context of brain maturation, we chose structural states to be scaled gray matter volumes in 26 bilateral regions of interest (ROI) covering large portions of the cortex and subcortical regions. We included up to two input variables as developmental factors driving the changes of the system (the framework can in general accommodate more than two such variables).

The input *u*_1_ was chosen to be an *a priori* fixed variable (also called manifest input) derived from puberty scores from boys and girls obtained from a separate modelling step (see Section 2.2 and Fig. 1B for details). The second input variable *u*_2_ was chosen to be a *latent or hidden* growth factor, which is modelled as an “alpha function” (Dayan and Abbott, 2001). It is equivalent to a second order differential equation (here expressed as two first order equations) (Grimbert and Faugeras, 2006)

(5) $$\begin{array}{l} \frac{{\mathrm{dv}}_{2}}{\mathrm{dt}}=-\frac{2}{\tau }v_{2}-\frac{1}{\tau^{2}}u_{2}+\frac{20}{\tau }\delta \\ \frac{{\mathrm{du}}_{2}}{\mathrm{dt}}=v_{2} \end{array}$$

where *δ* is a delta function at time zero (with $u_{2}(0)=1$ and $v_{2}(0)=0$) and *τ* denotes the peak response time of the hidden variable. In the context of our application to brain maturation, the model aims to describe the full evolution of gray matter in all ROIs over the full age range of the sample, i.e. from 6 to 22 years. Time zero here therefore refers to age of 6 years. The latent growth process then affects brain changes sooner or later during maturation, where the timing is flexibly modelled by parameter *τ* (see Fig. 1C top left for an illustration). This parameter also controls the duration, such that latent growth processes that peak later are also more enduring. It is important to note that although the dynamical system (in Eq. (4)) is of first order, the particular choice of hidden growth process (in Eq. (5)) implicitly defines a second order system, which includes accelerations, i.e. second temporal derivatives.

We note that the advantage of this particular choice of latent growth process is that dynamics are parsimoniously parametrised using a single additional parameter. However, implementing the growth factor using alpha dynamics might also be restrictive due to (1) having a fixed age of onset (at 6 years) and (2) potentially confounding variations of its delay with the duration of growth. Thus, firstly, we explored model comparisons of dynamic alpha growth with earlier and later age of onset. Secondly, we considered a more flexible alternative growth factor $u_{2}(t)=e^{-(t-m_{g})/(2\sigma^{2})}$ which has the shape of a Gaussian probability density function where we estimate one free mean parameter *m*_*g*_ for each of the gender groups and one independent free standard deviation parameter *σ*. We compare the empirical evidence for alternative model configurations including and excluding certain inputs using Bayesian model comparisons.

The differential equations governing the structural dynamics (Eq. (4)) and latent growth variable (Eq. (5)) are integrated using the (forward) Euler method with a time step of Δt=0.1 years. Initial gray matter states at time zero *x_i_*(0) are estimated during the overall optimisation of the model (for details see below). This produces a structural trajectory (i.e. time series) for each region *x*_*i*_. The predicted gray matter volumes are then given by

(6) $$g_{i}(\theta )=x_{i}$$

Therefore, the total set of parameters to be estimated are written as the vector $\theta =\{a_{\mathrm{ik}},c_{\mathrm{ij}},\tau ,x_{i}(0)\}$.

To summarize the structural dynamics parameters in the general case, for *D* brain regions we have *D* initial state values, *x_i_*(0), *D* regional self-connections *a*_*ii*_, and DX(D–1) between-region connection parameters *a*_*ik*_. For *J* (manifest or latent) input variables we then have DXJ regional sensitivity parameters *c*_*ij*_ and for each latent input variable one peak time variable *τ*_*j*_. Notably, some of the connection parameters or regional sensitivities might be *a priori* fixed to zero, rather than estimated. Thus, there is considerable flexibility in terms of implementing existing hypothesis about dynamics using the priors for all the parameters. For example, between-region dynamics might be omitted or restricted to allow only for interactions within specified subnetworks (as demonstrated in Section 3.4). It is important to note that the proposed modelling framework enables the investigation of *a priori* defined networks rather than inferring an unconstrained graph structure behind the connectivity matrix *A*. Moreover inputs can be constrained to act only upon certain regions of interest, e.g. with *a priori* known susceptibility to their effects.

The framework naturally accommodates structural dynamics for multiple subpopulations, e.g. gender or clinical groups. In order to do so, we introduced additional parameters describing group differences of *θ* and its components across different observed populations. Inference about group parameter differences, e.g. the question of whether the regional sensitivity *c*_*ij*_ is different between boys and girls, and any model comparisons involving group structures follow accordingly.

Finally, we aimed to compare the linear dynamical system to established cubic polynomial models which are independent across regions. In order to do so, the above generative model (Eqs. (4), (5), (6)) was replaced by a straightforward (state-independent) local cubic polynomials with the same noise model as used above, i.e.

(7) $$g_{i}(t,b)=\overset{3}{\underset{r=0}{\sum }}b_{\mathrm{ir}}t^{r}$$

with age or time *t*, coefficients *b*_*ir*_ for region *i* and polynomial order *r*.

### 2.5 Estimation and inference

In this framework we apply Bayesian inference for the inversion of the proposed dynamic models of structural change (for a general textbook introduction see (Bishop, 2006)). The prior specifications for the application to data on brain maturation are outlined in Appendix Section Appendix A. Generally priors are used to specify assumptions about the expected range of parameters and the general structure of the model. In this first proof of concept application of the framework we apply uninformative or weakly informative priors, which are aimed to represent data features in the parameters (given a certain model structure) rather than implementing strong and precise assumptions about their specific values.

We employ the Variational Laplace (VL) algorithm (Friston et al., 2007) for Bayesian parameter estimation, inference and model selection, specifically the implementation spm_nlsi_GN in release 6685 of SPM12 (Wellcome Trust Centre for Neuroimaging, London, UK, http://www.fil.ion.ucl.ac.uk/spm). The corresponding theory and algorithms have been introduced and validated in previous papers (Friston et al., 2007, Chumbley et al., 2007, Penny, 2012). A general mathematically detailed introduction to VL and the applied inference using model evidence is provided in Appendix B, Appendix C, Appendix D. Bayesian model comparison (Penny, 2012) is used to make inferences about basic modelling assumptions or gross features of the data. For example, whether to include any of the above considered external input factors in the dynamical model, whether boys and girls have the same sensitivities to the various growth factors, whether cubic polynomials perform comparably, or whether there are intrinsic interactions among brain regions.

It is important to mention that the dynamic MIMO input-state-output model presented in this article was fitted accounting for undesired confounds *x*_0_ in the observation model $y=g(x,w)+x_{0}b_{0}+e$. In particular, we included TICV to account for undesired effects of brain size variations, and site indicator variables to account for acquisition differences or other systematic differences between the six MRI scanning sites.

## 3 Results

### 3.1 Developmental factors as inputs

The proposed dynamic model incorporates two sources for non-linear changes of structural states. The first is the intrinsic connections reflecting inherent dynamics. In principle this means that the current state is affecting further evolution of the system. The second source is the effects of external inputs, here thought to be developmental factors or driving forces of development. Fig. 1B and C summarise the specifics of particular inputs considered in this study.

There is growing consensus that physiological changes during puberty affect the brain structure in specific ways. Our first input to the dynamical system was therefore defined as a fixed puberty factor. The particular temporal evolution of this factor was based on group-wise estimation of pubertal change curves using actual physiological pubertal status assessments provided by this NIH pediatric sample (see Fig. 1B). Sigmoidal fits (of Eq. (2)) revealed that the age of fastest changes of physical pubertal expression *p*_1_, was found to be 12.66 years (95%CI [12.49,12.84]) for girls and 14.34 years (95%CI [14.16,14.51]) for boys. This confirms expected gender-differences in timing and progression of puberty, which is later in boys relative to girls. The sigmoid slope parameters *p*_2_ were 0.21 (95%CI [0.19,0.23]) for girls and 0.22 (95%CI [0.20,0.24])for boys.

The second developmental factor we considered was intended to reflect a hidden (or latent) growth process, which causes tissue growth in early life. Hidden means that the actual driving force cannot be measured directly, but is rather inferred using the whole pattern of state progression in all ROIs. In particular, we chose an alpha function dynamic (see Eq. (5)) to cause gender-specific brain growth in all ROIs earlier or later during maturation (illustrated in Fig. 1C top left). The hidden growth factor dynamics are implicitly parametrised by including the unknown latency parameter *τ*, which is estimated from the data. Having a larger *τ* corresponds to a later and more enduring growth process. The estimation accounted for potential gender-group differences of this latency or delay parameter. In order to avoid potential bias due to the arbitrary choice of alpha growth dynamic onset being initially specified at an age of 6 years (the minimum age in the sample), we conducted a model comparison with varying age of onset, in particular using 3, 4, 5 or 7, 8, and 9 years instead. The results suggested highest model evidence for alpha growth dynamics with onset at age 5 years (Fig. 1D top). Therefore, all further dynamic modelling results are presented using this specific choice of early alpha growth with best empirical support in this particular sample. As suggested by our findings, the hidden growth appears to be later in boys compared with girls, indicated by the latency parameter *τ* (Fig. 1C top middle). The resulting shape of the hidden alpha growth factor (given the data) is shown in Fig. 1C top right.

In addition to the alpha dynamics, we also explored an alternative shape of the growth factor using a Gaussian. Compared with the alpha dynamics the Gaussian was chosen to be more flexible, having independent mean and standard deviation parameter (Fig. 1C bottom left and middle). Supporting the consistency of the above findings, the use of the Gaussian growth factor suggested a similar pattern of gender progression difference (Fig. 1C bottom right), with girls (with mean at 7.1 years) being affected earlier than boys (with 8.2 years) and a standard deviation of 2.2 years. Notably, we also observed similar trajectories and regional sensitivities using alpha and Gaussian growth dynamics (Fig. 1C right).

Furthermore, Bayesian model comparison was used to determine the input structure with the highest evidence in light of the data (see Fig. 1D bottom). In this comparison of inputs, we chose intrinsic dynamics arising from self-connections only (i.e. no interactions among regions, cf. Section 3.4). We compared models having: (1) no driving developmental factors at all; (2) only a puberty factor; (3) only a Gaussian growth factor; (4) a dynamic alpha growth factor; or (5) both puberty and alpha growth developmental factors. Notably, all factors were modelled to account for gender-differences. Although the single factors already increased the model evidence compared to considering only intrinsic dynamics, the highest evidence was found when combining both puberty and the alpha growth input. Thus, Bayesian model comparison suggested that the model fit improved even when accounting for increased model complexity including multiple factors. Finally, directly comparing the two considered candidates of growth factors in this sample, the dynamic alpha growth was found to have higher model evidence compared with the Gaussian parametric growth factor. The maximum evidence multivariate structural change model including puberty and alpha growth factors is further explored and specifically extended in the subsequent sections.

### 3.2 Trajectories, parameters and gender-differences

**Fig. 2:**
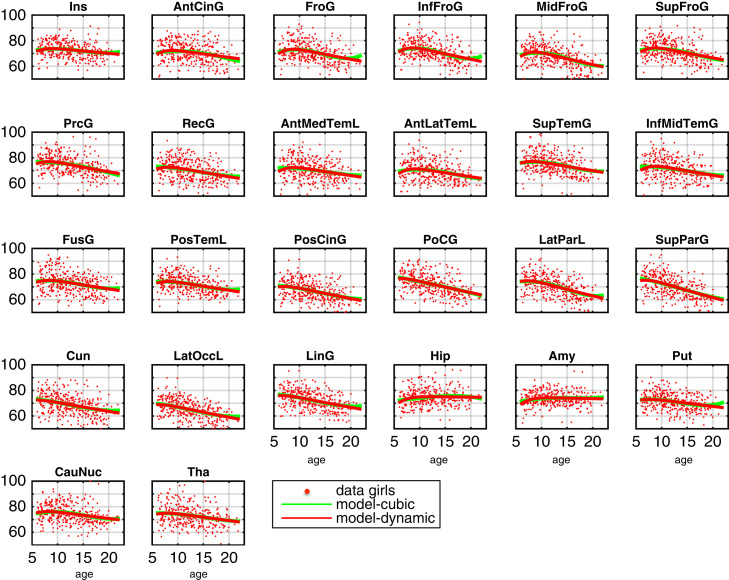
Girls developmental trajectories using uncoupled dynamical system with puberty-related factor and hidden growth factor. Dynamical systems model predictions and data for 151 girls is shown in red. Univariate cubic polynomial trajectory fits are shown in green.

**Fig. 3:**
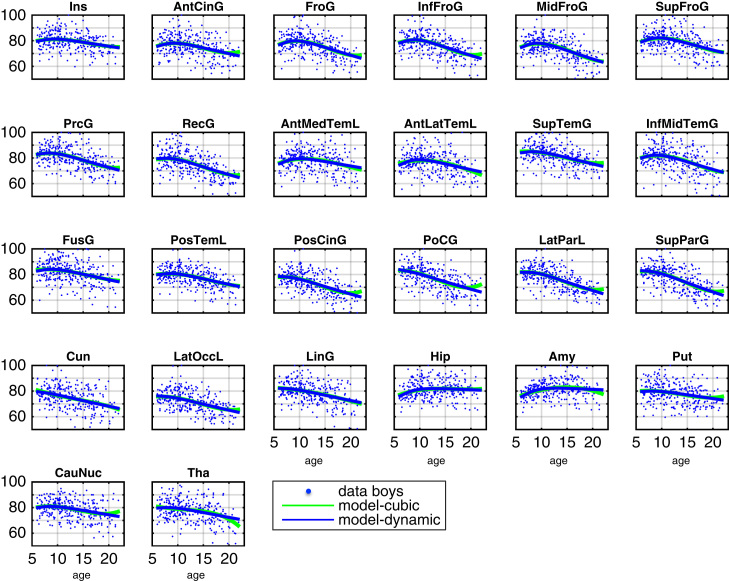
Boys developmental trajectories using uncoupled dynamical system with puberty-related factor and hidden growth factor. Dynamical systems model predictions and data for 135 boys is shown in blue. Univariate cubic polynomial trajectory fits are shown in green.

The complete data and dynamic model predictions in all bilateral ROIs are shown in Fig. 2, Fig. 3 for girls and boys respectively. The dynamic model accurately captures non-monotonic progression of the rescaled regional volumes. The inter-regional differences of progression e.g. cortical vs. subcortical, faster vs. slower decline appears flexibly modelled using the proposed method. Notably, as the main focus at this stage is the introduction of growth factors behind distributed patterns of change, the presented results so far are obtained from dynamical models containing only self-connections. However, this assumption will be relaxed in Section 3.4 below, where we explore examples of inter-regional connectivity in the intrinsic dynamics.

**Fig. 4:**
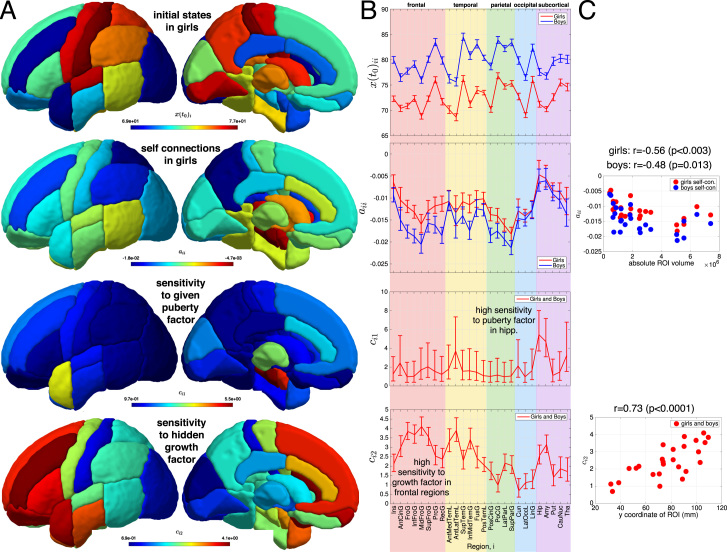
Overview of estimated dynamical system parameters using alpha growth and puberty input factors. A Posterior mean of initial state *x_i_*(0) (top), self-connections *a*_*ii*_ (top middle), and sensitivity parameters *c*_*ij*_ (bottom middle and bottom row) are shown using surface ROI projections. B Corresponding diagrams from parameters in A including error bars from posterior variance. Notably, the order of ROIs is adapted to follow brain lobes. C Relation of self-connection parameters to absolute volume size of the considered ROI (top) relation of alpha growth factor sensitivity to y coordinate on the anterior-posterior axis in the brains.

Since the proposed dynamical model inversion exploits Bayesian inference, we can further illustrate the model using posteriors from parameters of interest given the data (see Fig. 4). The obtained regional initial states and self-connection parameters are shown in Fig. 4A and B (top and top middle row). The values of the self-connections can be related to the time to lose *L* per cent of gray matter volume, via the formula *t_i_*(*L*)=(1/*a_ii_*) log(1–*L*/100). For example, a value of *a_ii_*= –0.005 (e.g. Hippocampus and Amygdala) indicates a loss of ten percent of gray matter in 21 years, whereas *a_ii_*= –0.02 (e.g. Middle Frontal Gyrus, Lateral Parietal Lobe) indicates the same loss in 5.3 years. The spatial pattern of self-connection values reveals regional differences of more or less rapid exponential decay of volumes, which goes beyond the effect of inputs. By ordering self-connections according to the size of the ROI, we observe an indication for larger regions showing stronger decay (Fig. 4C top). This is in line with the expectation that signals from larger ROIs are likely to have less variance and more noisy time series will be more influenced by the priors, which may cause them to show less decay.

The construction of our proposed dynamical system aims at understanding structural change as an effect of (observed or hidden) growth factors with potentially region-specific effects, e.g. due to variation in receptor density. Therefore the regional sensitivity/contribution parameters are of central interest for the validation of the model.

The sensitivity to the above specified pubertal and hidden growth factor is depicted in Fig. 4A and B (bottom middle and bottom row). Inspecting the posterior of sensitivity parameters, a strongest noticeable effect of the puberty factor to the regional structural progression was detected in subcortical regions, mainly hippocampus and amygdala ROIs. Compared to the overall effect of the hidden growth factor, the pubertal subcortical effects were rather small but spatially localised and distinctive.

More pronounced and widespread effects of the hidden growth factor were found. The posterior of the sensitivity to the growth factor was generally positive for all regions. However, the spatial pattern was indicative of the strongest contribution of a growth factor to the change in frontal and middle frontal gyrus. On a lobar level, frontal, temporal, parietal and occipital lobes exhibited decreasing sensitivity to the growth factor's effect, suggesting a substantial anterior-posterior gradient of childhood gray matter tissue growth (Fig. 4C bottom).

**Fig. 5:**
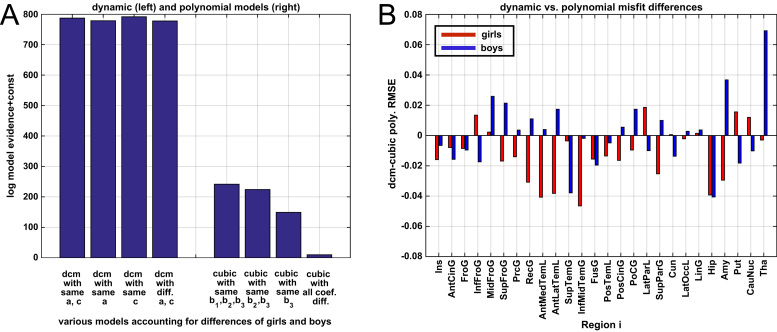
Model comparison of linear dynamical systems and cubic polynomials accounting for gender differences in multiple ways. Dynamical models included no gender-differences, or gender-differences for either connectivity *a*_*ik*_ or sensitivity *c*_*ij*_ parameters, or both. We compared multivariate linear dynamical systems including puberty and growth factors with independent cubic polynomials $\sum_{r=0}^{3}b_{r}t^{r}$ in all brain regions accounting for gender differences in different way by restricting coefficients *b*_*r*_ sometimes to be equal across gender groups. Bayesian model comparison is shown in A and root mean squared errors (RMSE) measure for all regions is shown for girls (red) and boys (blue) in B. Note that RMSE describes a measure of model fit not accounting for model complexity.

By construction, the initial structural state parameters were estimated on a group level capturing variability across genders (Fig. 4B top row). However, we explored whether there is also evidence for significant gender-differences of self-connections or sensitivity parameters. The results of Bayesian model comparison of four models that either impose similarity for these parameters across females and males is presented in Fig. 5A. Given our sample, the highest model evidence was found for a model with identical regional factor sensitivity but different self-connection parameters across genders. Notably, both included input factors were already accounting for gender in terms of age of strongest changes of pubertal expression and the latency of alpha growth. Therefore, using dynamical modelling our analysis revealed evidence for similar spatial patterns of regional sensitivity (to all considered developmental factors) for both genders.

### 3.3 Comparison with univariate cubic polynomials

We also aimed to compare dynamical systems-based multivariate trajectories with more established univariate methods. In particular, we modelled our data using an alternative univariate cubic polynomial (Eq. (7)) as a regionally independent generative model of the data instead of the proposed multivariate dynamical systems model. To avoid potential biases in this comparison, variance components, confounds, parameter estimation and model inference were completely consistent with the dynamical systems framework presented above. In order to additionally provide some flexibility for potential gender differences of the polynomial coefficients, we included four versions of this model where coefficients (1) *b*_1_, *b*_2_ and *b*_3_ or (2) *b*_2_ and *b*_3_ (3) only *b*_3_ or (4) no coefficient was supposed to be identical across both gender groups (with *b*_*r*_ referring to *b*_*ir*_ of region *i* in Eq. (7)). As a consequence considering models (1)-(4) have a varying number of parameters and provide some flexibility for fitting the data. It is important to note that the polynomial model fits local functions of time without any dependencies of coefficients across brain regions while the multivariate dynamical systems considered in this section predicts change based on a common underlying growth factor.

The resulting univariate polynomial trajectories can be seen together with the multivariate dynamical systems results in Fig. 2, Fig. 3 for girls and boys respectively. Numerical estimates of ages of peak volume in all regions are provided in Table 1 of the Supplementary material. From visual inspection, trajectories obtained using both differing methods were very similar. However, because we specified the two types of model as generative models for all brain regions in the same framework, this also allowed us to perform formal Bayesian model comparison of dynamical versus cubic polynomial model (see Fig. 5A).

Using our sample, model evidence uniformly favoured dynamical systems compared to the univariate polynomials accounting for potential differences between girls and boys. Since there are no widely established methods for analysis of coupled structural changes in developmental trajectories, this comparison provides a coarse indication of the face validity of our novel approach. We further compared the regional root mean square of errors (RMSE) from the highest evidence model of each class of models, which is shown for girls and boys in Fig. 5B. The obtained RMSE also did not indicate systematically better or worse fit for either of the models across multiple brain regions. This was supported by a two-sample F test for equal residual variances in each region, which did not show significant deviations (all p values >0.4). Taken together, our analysis suggested that the linear dynamical system trajectories did not show significantly worse fit and entailed lower model complexity leading to higher model evidence. Notably, although very flexible, the polynomials do not directly support a potential mechanistic interpretation of observed structural changes or enable testing specific ideas about the underlying generative process across brain regions.

### 3.4 Including inter-regional dynamics

In the previous sections we presented dynamic models of structural changes, which contained external inputs and internal self-connections. Regions were assumed to develop independently and were ’coupled’ only by being affected by the same driving input. However, there are physical and neuroscientific arguments for including explicit interactions among regional states in the dynamics. Finally, we aimed to demonstrate the potential of the framework for comparing more complex patterns of intrinsically connected structural changes in development. More specifically, we explored the hypothesis that inter-regional dynamics during brain development would be stronger *within* the same module of co-activation than *between* different functional modules as observed in resting state fMRI (rsfMRI).

**Fig. 6:**
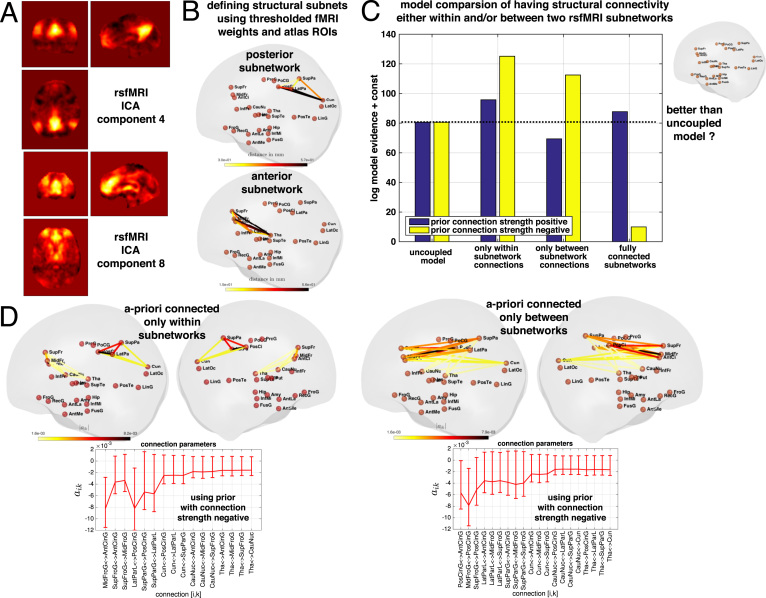
Comparing structural change models including inter-regional dynamics based on *a priori* defined rsfMRI subnetworks. A Resting state ICA component weight maps 4 and 8 available from a set of 10 selected maps, provided as supplementary material in Smith et al. (2009). B Data-driven definition of two subnetworks of ROIs analyzed in our study including regions with highest ICA component weights averaged within ROIs. The euclidian distance of all included nodes (ROIs) in each subnetwork is shown. C Bayesian model selection of connectivity models comparing log model evidence (in 289 subjects sample) of four models including (1) no inter-regional dynamics; (2) only connections within each of the two subnetworks; (3) only connections between the two subnetworks; or (4) fully connected subnetworks containing all possible connections (not shown). All models were considered with weakly informative log-normal priors constraining connection strength to be either only positive or only negative. D Illustration and diagrams of absolute connection strength *a*_*ik*_ of the two highest evidence models from model comparison. Only undirected networks were explored, i.e. we set a further constraint to use symmetric matrices $a_{\mathrm{ik}}=a_{\mathrm{ki}}$ for all considered networks.

We here built on a previous study identifying major intrinsic connectivity networks in the brain, as imaged with rsfMRI in 36 subjects (Smith et al., 2009). The authors provided independent component analysis (ICA) maps in standardized space as supplementary material, including ten components from rsfMRI that could be well matched with corresponding components from task fMRI (http://fsl.fmrib.ox.ac.uk/analysis/brainmap+rsns/PNAS_Smith09_rsn10.nii.gz). As an example, we here focussed on the posterior and anterior co-activation networks provided in component 4 (default mode network) and component 8 (executive control network) of their analysis respectively (see Fig. 6A). For each of these two component maps, we identified the set of brain regions containing the highest average ICA co-activation weights within the available 26 gray matter ROIs analysed in previous sections. To include the major nodes within each co-activation network observed in resting state, we further considered only ROIs showing an average component weight above 40% of the maximal averaged component weight found in all ROIs. This procedure resulted in (A) an anterior subnetwork including the ROIs of anterior cingulate, middle and superior frontal gyrus, caudate and thalamus; and (B) a posterior subnetwork including ROIs of posterior cingulate cortex, cuneus, lateral parietal lobe and superior parietal gyrus (illustrated in Fig. 6B).

We implemented the above hypothesis by focussing on Bayesian model selection among models having (1) no coupling between all ROIs; (2) coupling only *within* the two subnetworks; and (3) coupling only *between* the subnetworks; and (4) full coupling of all ROIs from both subnetworks (see an illustration of (2) and (3) in Fig. 6D). In particular, models (2)-(4) were compared having either only positive or negative connections strength. Note also, that reciprocal connections were considered, i.e. the connectivity matrix was assumed to be symmetric *A*=*A^T^* .

If there is substantial inter-regional dynamics in terms of connection parameters in the considered (sub-)networks, one would expect a higher model evidence of a coupled compared with an uncoupled model. In fact, the results of our model comparison suggested inter-regional structural dynamics during brain maturation with especially negatively connected models showing a higher model evidence (Fig. 6C). A negative connection strength means that higher regional volume is associated with more shrinkage (or less growth) of the connected brain region (see estimates in Fig. 6D). Moreover, results indicated that the model evidence was higher when the (negative) structural connectivity did reflect the pattern of functional co-activation. This was evident since we found a higher model evidence for models including connections *within* subnetworks rather than when including structural connections *between* subnetworks (reflecting co-activations in rsfMRI in (Smith et al., 2009)).

## 4 Discussion

In this work we address dynamic and non-linear aspects of structural brain changes typically observed in longitudinal MRI studies on development. Unlike more traditional approaches, e.g. using the general linear model or linear-mixed effects, in which flexible models are fitted independently to multiple brain regions, we here propose a framework for modelling concerted change in terms of a multivariate dynamical model. This goes beyond existing approaches by avoiding limitations of mass-univariate testing and incorporating a mechanistic perspective on dynamic phenomena during normal and pathological brain development. The model not only describes the evolution of structural states (accounting for potential interactions) but also allows inputs or driving forces to be specified, which can represent e.g. the presence of certain growth factors, toxins, proteins etc. In the current form, the framework enables formulating parsimonious multivariate models of brain growth typically seen in studies on development (Mills and Tamnes, 2014). As demonstrated in a developmental dataset, Bayesian model comparison affords investigation of specific hypotheses about effects of developmental factors or inter-regional dynamics.

The approach is motivated by the ambitious longterm goal of progressing towards mechanistic or process-oriented models that implement specific neuroscientific hypotheses, rather than using explorative analysis with *post hoc* integration of results (Stephan et al., 2015, Montague et al., 2012). Explicit specification of growth factors is also reflective of an ongoing trend towards increased availability of physiological measurements from blood serum, cerebrospinal fluid, gene expression atlases, or other imaging modalities in large sample studies, projects and initiatives (e.g. the Allen Brain Atlas, http://www.brain-map.org/). The mechanistic perspective might be useful to study the rapid growth of the human cortex during development, especially the cortical folding of the brain into a highly convoluted structure in fetuses and newborns (Lefèvre et al., 2015). Moreover, there is evidence that, although molecular determinants control tangential expansion of the cortex, the size, shape, placement and orientation of the folds might arise through principles of mechanical instability modulated by fetal brain geometry (Tallinen et al., 2016).

In this work on modelling changes in sMRI datasets, we employ estimation and inference algorithms introduced earlier (Friston et al., 2007). These procedures have been validated for dynamical systems in context of functional and electrophysiological brain data (Daunizeau et al., 2009). General issues on the identifiability of dynamic models have been previously studied in context of effective connectivity (Valdes-Sosa et al., 2011, Arand et al., 2015).

This paper focussed on demonstrating construct validity for structural dynamical modelling using longitudinal data from brain maturation in 289 subjects from the NIH paediatric repository. For this purpose, we specified a system having intrinsic linear dynamics with state vectors representing gray matter volume in a whole brain network and two types of inputs or driving forces. It is worth mentioning that including explicit growth factors in the model of structural development extends most existing work by (a) dynamic modelling of change and the sources of variability beyond time and (b) modelling or inferring (hidden) causes to facilitate mechanistic interpretations.

In order to explore the validity of our new modelling approach we compared different generative models using Bayesian inference and model selection. Findings consistently suggested higher model evidence and similar model fit when comparing the proposed multivariate dynamical model with established univariate cubic polynomials often used in developmental samples. Given the advantage of this novel framework to implement mechanistic and spatially multivariate hypotheses, this suggests that dynamical systems might be a promising approach to model brain changes in development and aging.

More specifically, to demonstrate that *a priori* known inputs can be integrated into the model, we derived a puberty-related factor based on available physical assessments in the maturation sample. Model comparisons suggested a significant contribution of the included puberty factor. The posterior distribution over model parameters indicated highest sensitivity within the hippocampus-amygdala complex for the puberty factor's effects. The hippocampus has been repeatedly implicated in learning, memory and cognition (Zeidman and Maguire, 2016). More specifically, the same authors proposed that the involvement of the hippocampus in visual perception, imagination and episodic recall might be related to the process of learning and updating models of the world. Thus, we speculate that our findings of puberty-related changes might mark the beginning of the reduction of underlying learning rates during updating the individual models (of the world). Our volumetric findings are also in line with other recent studies showing effects of pubertal Tanner staging or hormonal data in the hippocampus (Goddings et al., 2014, Brouwer et al., 2015, Herting et al., 2014, Giedd et al., 2006). The first factor's effects can be seen as a sign of validity of our proposed dynamic modelling approach. Notably, the observed puberty-induced changes were found to be small compared to overall developmental effects during the explored age range.

The proposed framework goes beyond integrating well known (or assumed) inputs or driving forces and motivating mechanistic interpretations on brain changes. We show that this framework allows inferring hidden developmental factors that are likely to cause the observed patterns of state change in multiple brain regions or whole networks. This inference on hidden causes is a central contribution of this work. Firstly, since more modalities and physiological parameters become increasingly available in large neuroimaging projects, quantitative analysis of correlated multivariate change indices might be best performed by characterising the underlying causal interactions at the latent/hidden variable level. Secondly, having established latent causes for growth or atrophy in healthy and diseased aging samples would allow more powerful predictions for future time points, even in common practical situations with sparse observations, missingness and very noisy data.

Since the gray matter volume features in the analysed maturation sample indicated a non-monotonic (inverse U-shaped) development in some brain regions, we aimed to model the underlying growth process in a dynamic fashion. Crucially, it has recently been proposed that childhood cortical thickness growth in developmental studies might be artifactual due to movement, image quality or brain size variations (Ducharme et al., 2016). Since our focus is on the modelling approach rather than phenotype-specific questions of the brain features, we aimed to rule out the possibility of biased results by applying state of the art longitudinal morphometry processing techniques, rigorous quality control and inclusion of brain size and scanning site variations as between-subjects confounds in the model inversion. We cannot exclude that unnoticed movement artefact differences might have contributed to our finding of a childhood gray matter volume growth. However, this is unlikely because the growth was found to be region-specific in anterior and prefrontal gray matter regions, rather than a global effect. In addition, voxel-based segmentations of regional gray matter volumes also reflect changes in surface area and were found to show more curvilinear trajectories than surface-based cortical thickness features (Raznahan et al., 2011, Wierenga et al., 2014).

In particular, we explored dynamics of hidden factors driving change of regional gray matter in females and males. According to recent evidence showing that gray matter exhibits its highest volume during mid-to-late childhood (Tamnes et al., 2013, Aubert-Broche et al., 2013, Wierenga et al., 2014, Mills et al., 2016) the growth factor was initially modelled as beginning to affect the brain at age six and with a free delay/dispersion parameter for females and males. This particular choice for the age of onset of the driving forces was motivated by the given age range of the dataset rather than biological arguments. Although clearly restricted to the considered ages, during model selection, a parametrisation with slightly earlier onset of growth at age 5 years showed higher model evidence. Including an alpha dynamic growth factor generally improved the model evidence and the posterior of the latency parameter indicated a delayed tissue change process in males compared to females. One might also argue that the general shape of the alpha dynamic growth might be too restrictive, and thus leading to biased findings. However, exploring an alternative implementation of growth using a parametric Gaussian ’growth impulse’ with more flexible shapes also suggested a similar pattern of gender-specific progression difference. This is supported by recent work using a multivariate brain development index (BDI) indicating developmental time-lags between genders in late childhood and early adolescence (Erus et al., 2015). The consistency of our findings and previous findings is encouraging and supports the validity of this newly presented framework.

In our analysis, the model with highest evidence contained multiple combined growth factors, in particular, the static puberty factor and hidden alpha growth. Moreover, modelling the (hypothesized) driving forces of development in the state equations directly (e.g. as alpha dynamics) outperformed feeding parametric curves (e.g. Gaussian with mean and sd) as inputs to the system. This should be further explored in future applications and suggests that extending and adapting the set of (parametric and dynamic) growth factors for various scenarios would be fruitful.

Interestingly, the estimated regional values of sensitivity/contribution of both explored hidden growth factors showed a consistent anterior-posterior gradient with strongest impact on prefrontal cortex (PFC) brain changes. This is in line with some MR-based studies showing that the human PFC undergoes protracted macro-structural changes in the form of inverted U-shaped trajectories with childhood growth followed by decline throughout adolescence and early adulthood (Shaw et al., 2008, Group, 2011). The observed macro-anatomical model findings might be related to fact that the human PFC also undergoes a prolonged phase of microstructural reorganization with involved processes of cortical myelination and synaptic changes (Tau and Peterson, 2009; Petanjek et al., 2011).

There is evidence, that neurotrophic growth factors might influence the differentiation and survival of neurons and glia cells, and substantially modulate synaptic changes (Tau and Peterson, 2009). Although we demonstrated inferring potential causes for tissue growth in terms of hidden growth factors, our dataset did not include any physiological or serum parameters, hormones, neurotransmitters or neurotrophins from the analysed sample. We therefore cannot conclusively infer what actually caused the observed changes in the participants’ brains. However, as shown for the puberty scores, the framework allows inclusion of physiologically informative inputs when available in future work. For example, it would be possible to include actual observed hormonal data and/or metabolic parameters in the same sample. Thus, we can only speculate that neurotrophins, such as the brain derived neurotrophic factor (BDNF), might be involved in the physiological processes underlying the hidden growth factor.

We conducted model comparisons of two previously observed network-modules of functional co-activation using resting state fMRI (Smith et al., 2009). The results suggest that there are substantial inter-regional dynamics during brain maturation in terms of significant negative couplings across regions in the explored space of models. Focussing on these particular subnetworks and the coarse parcellation into 26 bilateral gray matter ROIs, our findings suggest that inter-regional coupling of structural changes (during brain maturation, late childhood and adolescence) might follow patterns of functional co-activation or independence respectively. Since parametrising dynamics in this way has not been reported yet, this finding shows the potential of the approach to go beyond existing work. We cannot exclude the possibility that local coupling might be partially influenced by local optima in the image registration. However, if local registration errors caused couplings of nearby regions, the direction of coupling would also vary from negative to positive, which was not observed in our analysis. Taken together our findings suggest that exploring the nature of the structural dynamics, for example by combining morphometry with diffusion data in future developmental studies, might be a promising direction of research (see also Stephan et al., 2009).

The above provided signs of validity about (A) introducing and comparing explicit growth factors into models of brain changes and (B) capturing inter-regional dynamics might offer a way forward for studying causes of structural covariance (Mechelli et al., 2005) and correlated structural changes (Raznahan et al., 2011). Moreover, there has been substantial progress in modelling disease progression in multiple imaging and clinical biomarkers using an event-based perspective (Fonteijn et al., 2012, Young et al., 2014, Sabuncu et al., 2014). Event-based approaches share some motivation with our approach because both aim at generative modelling of complex multivariate processes over time. However, while dynamical systems approaches model progression in the form of forces or interactions across modalities or brain regions, event-based models often rather aim at inference about the sequence/order rather than the parameters of mechanics most likely to cause them.

In the study of degenerative diseases, estimation of dynamical systems parameters would permit inferences about how quickly neuronal degeneration in one region propagates to another. One might hope to relate such parameters to the time constants of underlying putative molecular processes. For example, in Alzheimer's, how quickly pathology is spread via trans-synaptic transmission of mutated tau protein (Ballatore et al., 2007) or how quickly synaptic dysfunction in one region begets synaptic dysfunction in another (Newman et al., 2012). There is growing interest in how general proteinopathies damage complex brain networks (Warren et al., 2013). Notably, there was a recent attempt to explore dynamical systems for disease progression in Alzheimer's Disease (AD) (Oxtoby et al., 2014). The study focussed on ventricle expansion in pathologically aging clinical groups while accounting for potential covariates in the progression. Here we extend this idea to model structural progression dynamics in multiple regional biomarkers using an explicitly multivariate generative modelling framework. Moreover, our approach additionally incorporates inter-regional network connectivity, which might affect the progression within the nodes. Future work might focus on dynamics in the presence of detrimental physiological factors in neurodegenerative disease.

We would finally like to mention further limitations and potential extensions for future work. In this early attempt at dynamic modelling of sMRI, the observation model was focussed on well established macro-anatomical morphometric markers. While pure morphometric results are important and encouraging, they might lack a certain degree of physiological specificity. It might be also worth applying the dynamical model to features other than volume, such as cortical thickness, local gyrification, or surface area growth. In future, we aim to overcome this by augmenting the current trivial observation model to accommodate data from multiple imaging sequences in a joint biophysical forward model, to allow exploration of state changes of *in vivo* histological parameters, for example local myelination (Weiskopf et al., 2015).

Another assumption that warrants revisiting in future work derives from the fact the self-connections are constrained to be negative. This means that in the absence of external perturbation (e.g. from hormonal or latent growth factors) gray matter density will decrease. An alternative model assumption could have been that the self-connections are positive i.e. that regional gray matter will increase unless constrained by external perturbation (e.g. competition for space). Whilst we did informally explore some models of this nature (not reported) our experience led us to conclude these models were inherently unstable. Nevertheless, a more thorough investigation may be of interest in the future.

It is important to note that, here, we aimed to model longitudinal dynamics at the level of groups or populations, rather than single individuals. While the individual differences of change in expression of puberty and brain structure are neglected in the current formulation of our dynamic model, they would need to be addressed in context of highly variable process of healthy and pathological ageing. Future work may make use of recent methodological advances that have the potential for additional sources of heterogeneity to be accounted for within dynamical systems (Penny and Sengupta, 2016, Sengupta et al., 2016, Friston et al.,).

## Appendix A Applied priors, likelihood and variance components

Here we outline the specifics of the applied model priors for the inversion of dynamical models of structural changes in the brain maturation sample. The following prior specification considers the special case of models including multiple driving inputs, i.e. puberty and alpha growth, but without having inter-regional coupling (in 3.1, 3.2, 3.3 above), where off diagonal connections are assumed to be zero, i.e. $a_{\mathrm{ik}}=0$, for i≠k. The uninformative or weakly informative priors on the free parameters p(θ|m), were specified in terms of expectation and covariance,

(8) $$p(\theta |m)=p(\tau )\overset{D}{\underset{i=1}{\prod }}\left[p(a_{\mathrm{ii}})p(x_{i}(0))\overset{J}{\underset{j=1}{\prod }}p(c_{\mathrm{ij}})\right]\;p(a_{\mathrm{ii}})=a_{0}\exp({\overset{˜}{a}}_{\mathrm{ii}}),\;\mathrm{with}\;p({\overset{˜}{a}}_{\mathrm{ii}})=N(0,1)\;p(x_{i}(0))=N(\mu_{i},64)\;p(\tau )=\tau_{0}\exp(\overset{˜}{\tau }),\;\mathrm{with}\;p(\overset{˜}{\tau })=N(0,1)\;p(c_{\mathrm{ij}})=c_{0}\exp({\overset{˜}{c}}_{\mathrm{ij}}),\;\mathrm{with}\;p({\overset{˜}{c}}_{\mathrm{ij}})=N(0,1)$$

where *m* indexes model assumptions (e.g. tied parameters for boys and girls), $a_{0}=-0.0066$, $\tau_{0}=2$ and $c_{0}=2$. In particular, *a*_0_ was chosen so that the *a priori* most likely time constant produced a decay to 0.9 of the initial value (the value at time zero) within the time period under study. In this application the applied time-constant is constrained to be negative since the coarse trend in the data is volumetric reductions and a positive time-constant would require state equations with additional constrains which prevent growth from becoming instable. Sensitivity parameters *c* and alpha dynamic parameter *τ* were also specified using log-normal priors allowing for substantial or almost no contribution of the considered inputs and for flexible developmental delays/time-lag of growth during middle and later childhood respectively. Here *μ*_*i*_ refers to the mean of the first five observations for the *i*th state variable. Priors on initial values $x_{i}(0)$ were chosen to have very large variances, to be rather uninformative.

Alternatively, when exploring the latent parametric Gaussian instead of the dynamic alpha function growth input, we applied the following prior on parameters

(9) $$\;p(\theta |m)=p(m_{g})p(\sigma )\overset{D}{\underset{i=1}{\prod }}\left[p(a_{\mathrm{ii}})p(x_{i}(0))\overset{J}{\underset{j=1}{\prod }}p(c_{\mathrm{ij}})\right]\;p(a_{\mathrm{ii}})=a_{0}\exp({\overset{˜}{a}}_{\mathrm{ii}}),\;\mathrm{with}\;p({\overset{˜}{a}}_{\mathrm{ii}})=N(0,1)\;p(x_{i}(0))=N(\mu_{i},64)\;p(m_{g})=N(8,100^{2})\;p(\sigma )=N(5,100^{2})\;p(c_{\mathrm{ij}})=c_{0}\exp({\overset{˜}{c}}_{\mathrm{ij}}),\;\mathrm{with}p({\overset{˜}{c}}_{\mathrm{ij}})=N(0,1)$$

with same fixed *a*_0_ and *c*_0_ parameters as used above, and prior mean age of the Gaussian growth was set to 8 years for boys and girls and large prior variance to provide substantial flexibility.

We also analysed inter-regional dynamics during brain maturation presented in Section 3.4 where in addition to the main-diagonal connectivities *a*_*ii*_ we allowed off-diagonal connectivity parameters *a*_*ik*_ (for i≠k) of certain networks (specified above) to have non-zero values. More specifically, the applied priors in the case of a fully connected network with complex intrinsic dynamics follows as

(10) $$\;p(\theta |m)=p(\tau )\overset{D}{\underset{i=1}{\prod }}\left[p(x_{i}(0))\overset{D}{\underset{k=1}{\prod }}p(a_{\mathrm{ik}})\overset{J}{\underset{j=1}{\prod }}p(c_{\mathrm{ij}})\right]\;p(a_{\mathrm{ii}})=a_{0}\exp({\overset{˜}{a}}_{\mathrm{ii}}),\;\mathrm{with}\;p({\overset{˜}{a}}_{\mathrm{ii}})=N(0,1)\;p(a_{\mathrm{ik}})=b_{0}\exp({\overset{˜}{b}}_{\mathrm{ik}}),\;\mathrm{with}\;p({\overset{˜}{b}}_{\mathrm{ik}})=N(0,1),i\neq k\;p(x_{i}(0))=N(\mu_{i},64)\;p(\tau )=\tau_{0}\exp(\overset{˜}{\tau }),\;\mathrm{with}\;p(\overset{˜}{\tau })=N(0,1)\;p(c_{\mathrm{ij}})=c_{0}\exp({\overset{˜}{c}}_{\mathrm{ij}}),\;\mathrm{with}\;p({\overset{˜}{c}}_{\mathrm{ij}})=N(0,1)$$

using the same *a*_0_, *τ*_0_ and *c*_0_ as specified previously. More specifically, we here used log-normal priors on the inter-regional connectivities to constrain interactions and compare models with certain networks having only positive (*b*_0_=0.005) or only negative ($b_{0}=-0.005$) connections strengths.

Finally, in the model comparison of multivariate dynamical systems with regionally independent cubic polynomials (Eq. (7)) we specified weakly informative priors as follows

(11) $$p(\theta |m)=\overset{D}{\underset{i=1}{\prod }}\overset{3}{\underset{r=0}{\prod }}p(b_{\mathrm{ir}})\;p(b_{i0})=N(\mu_{i},10^{2})\;p(b_{\mathrm{ir}})=N(0,10^{2-r}),\;r=1,2,3$$

with coefficients *b*_*ir*_ of region *i*, polynomial degree *k* and *μ*_*i*_ the mean of the observations in region *i*.

Furthermore, for all considered generative models above (e.g. dynamic alpha growth, parametric Gaussian growth, and independent cubic polynomials), the likelihood of the data under model *m* was

(12) $$p(y|\theta ,\lambda ,m)=N(g(\theta ),C_{e})\;C_{e}=\left(\underset{i}{\sum }\lambda_{i}Q_{i}\right)+\lambda_{r1}Q_{r1}+\lambda_{r2}Q_{r2}$$

where the data vector *y* comprises (vectorised) *D* dimensional structural measures at all time points from all subjects, g(θ) are (vectorised) model predictions, and *C*_*e*_ is an error covariance matrix composed of a sum of weighted covariance functions, *Q*_*i*_, as described below. Here, *y* and g(θ) are vectorised over subjects in a consistent manner (i.e. all from subject 1, all from subject 2. etc). The covariance component weights *λ* and model parameters *θ* are estimated using VL (Friston et al., 2007).

As the model is specified at the group level, as opposed to the level of individuals, the repeated measures design induces correlated residuals within subject. In order to enable unbiased inference, these correlations need to be accounted for in terms of a covariance structure, *C*_*e*_, which here was adapted to the specifics of the given accelerated longitudinal design of the study. We used the following set of covariance basis functions to model the main diagonal and additionally some specific off-diagonal elements of *C*_*e*_. We specified *Q*_*i*_ for *i*=1,..., *D* to have only sparse entries of ones on the main-diagonal for region *i*, if the corresponding entry in observation vector *y* is from a girl. Specifying variance components *Q*_*i*_ for *i*=*D*+1,...,2*D* correspondingly for boys results in hyper-parameter *λ*_*i*_ capturing the error variance of all observations independently for both genders and also separately for all brain regions. Additionally, because the imaging sample contained 2–3 annual scans per subject, we specified one further variance component *Q*_*r1*_ having only specific non-zero off-diagonal entries (of one) if the particular observation was measured 1 year apart of another observation for the same subject. As a consequence, hyper-parameter *λ*_*r1*_ becomes the pooled estimate (across all subjects in the sample) of the covariance of observations for a one-year time difference. Similarly, *Q*_*r2*_ and *λ*_*r2*_ were used to capture potential two-year time difference correlations.

## Appendix B Variational laplace

In what follows below N(x;m,C) denotes a multivariate normal distribution with mean *m* and covariance *C*. The Variational Laplace (VL) algorithm (Friston et al., 2007) can be used for Bayesian estimation of any nonlinear model of the form

(13) y=f(θ,m)+e

where f(θ) is a nonlinear function specified by model *m*, and *e* is zero mean additive Gaussian noise with covariance *C*_*y*_. This covariance depends on hyperparameters *λ* as shown below. The likelihood of the data is therefore

(14) $$p(y|\theta ,\lambda ,m)=N(y;f(\theta ,m),C_{y})$$

The framework allows for Gaussian priors over model parameters

(15) $$p(\theta |m)=N(\theta ;\mu_{\theta },C_{\theta })$$

where the prior mean and covariance are assumed known. The error covariances are assumed to decompose into terms of the form

(16) $$C_{y}^{-1}=\underset{i}{\sum }\exp(\lambda_{i})Q_{i}$$

where *Q*_*i*_ are known precision basis functions. The ‘noise parameters’ or hyperparameters that govern these variance components are collectively written as the vector *λ*. These will be estimated. Additionally, the hyperparameters are constrained by the prior

(17) $$p(\lambda |m)=N(\lambda ;\mu_{\lambda },C_{\lambda })$$

The above distributions allow one to write down an expression for the joint probability of the data, parameters and noise parameters

(18) p(y,θ,λ|m)=p(y|θ,λ,m)p(θ|m)p(λ|m)

The starting point for variational inference is then to assume a factorisation of the posterior density (Beal, 2003). The VL algorithm is based on the assumption that the approximate posterior density has the following factorised form

(19) $$q(\theta ,\lambda |y,m)=q(\theta |y,m)q(\lambda |y,m)\;q(\theta |y,m)=N(\theta ;m_{\theta },S_{\theta })\;q(\lambda |y,m)=N(\lambda ;m_{\lambda },S_{\lambda })$$

Importantly, the factorisation is between parameters and noise parameters only. Dependencies among model parameters are explicitly accounted for in the posterior covariance matrix *S*_*θ*_. For a model with *p* parameters *S*_*θ*_ is a [p×p] matrix.

## Appendix C Model parameters

The above distributions allow one to write down an expression for the joint log likelihood of the data, parameters and hyperparameters

(20) L(θ,λ)=log[p(y|θ,λ,m)p(θ|m)p(λ|m)]

To make a connection with statistical physics we define the ‘energy’ as the negative log joint, –*L*. Thus high probability parameters correspond to low energy states. For the Gaussian likelihood and priors defined above we have

(21) $$L(\theta ,\lambda )=-\frac{1}{2}e_{y}^{T}C_{y}^{-1}e_{y}-\frac{1}{2}\log|C_{y}|-\frac{N}{2}\log2\pi \;\;-\frac{1}{2}e_{\theta }^{T}C_{\theta }^{-1}e_{\theta }-\frac{1}{2}\log|C_{\theta }|-\frac{p}{2}\log2\pi \;\;-\frac{1}{2}e_{\lambda }^{T}C_{\lambda }^{-1}e_{\lambda }-\frac{1}{2}\log|C_{\lambda }|-\frac{h}{2}\log2\pi$$

with *N* data points, *p* parameters, *h* noise parameters and error terms given by

(22) $$e_{y}=y-f(\theta ,m)\;e_{\theta }=\theta -\mu_{\theta }\;e_{\lambda }=\lambda -\mu_{\lambda }$$

The negative “variational energies” with respect to parameters and noise parameters are then defined as (see Eq. (4) in Friston et al. (2007) for generic form, or a tutorial treatment leading up to Eq. (6) in the ‘Variational Bayes’ chapter of Friston et al. (2007))

(23) I(θ)=∫L(θ,λ)q(λ|y,m)dλI(λ)=∫L(θ,λ)q(θ|y,m)dθ

If *D*_*θ*_ and *D*_*λ*_ are matrices of second order derivatives of *L* with respect to *θ* and *λ* (i.e. Hessian matrices) then the variational energies can be evaluated as

(24) $$I(\theta )=L(\theta ,m_{\lambda })+\frac{1}{2}\mathrm{Tr}(S_{\lambda }D_{\theta })\;I(\lambda )=L(m_{\theta },\lambda )+\frac{1}{2}\mathrm{Tr}(S_{\theta }D_{\lambda })$$

See Eq. (6) in Friston et al. (2007) for generic form. The parameters of the approximate posteriors, $\left\{m_{\theta },S_{\theta },m_{\lambda },S_{\lambda }\right\}$, are then iteratively updated so as to minimise these variational energies. This is mathematically equivalent to minimising the Kullback-Liebler divergence between the true and approximate posteriors (Beal, 2003, Friston et al., 2007).

This maximisation is effected by first computing the gradient and curvature of the variational energies at the current parameter estimate, $m_{\theta }(\mathrm{old})$. For example, for the parameters we have

(25) $${\;j}_{\theta }(i)=\frac{\mathrm{dI}(\theta )}{d\theta (i)}\;H_{\theta }(i,j)=\frac{d^{2}I(\theta )}{d\theta (i)d\theta (j)}$$

where *i* and *j* index the *i*th and *j*th parameters, *j*_*θ*_ is the gradient vector and *H*_*θ*_ is the curvature (Hessian) matrix. The estimate for the posterior mean is then given by

(26) $$m_{\theta }(\mathrm{new})=m_{\theta }(\mathrm{old})+\Delta m_{\theta }$$

where

(27) $$\Delta m_{\theta }=\left[\exp({\mathrm{vH}}_{\theta })-I\right]H_{\theta }^{-1}j_{\theta }$$

This last expression implements a ‘temporal regularisation’ with regularisation parameter *v* (Friston et al., 2007) (so-called, because Eq. (27) is the solution to the differential equation $\frac{d\theta }{\mathrm{dv}}=H\theta$ at ‘time’ point *v*). In the limit v→∞ the update reduces to

(28) $$\Delta m_{\theta }=-H_{\theta }^{-1}j_{\theta }$$

which is equivalent to a Newton update (Press et al., 1992). This implements a step in the direction of the gradient with a step size given by the inverse curvature. Big steps are taken in regions where the gradient changes slowly (low curvature).

In regions of parameter space near maxima the curvature is negative definite (hence the sign in Eq. (28)). However, in other regions this is not necessarily the case and the curvature should not be fully trusted. This is the motivation behind model trust-region approaches (Press et al., 1992) and temporal regularization falls into this category of algorithm. Additionally, for the parameters, an adaptive regularisation is used. If the update in Eq. (27) does not result in a decrease in variational free energy (see next section) then *v* is reduced by 1/2. If it does, then *v* is increased by 1/2 (the curvature becomes more trustworthy). In addition *v* is constrained to lie between -4 and +4. This algorithm was found to provide more robust optimisation than standard Newton approaches on a variety of optimisation problems (see e.g. Figure 1 in Friston et al., 2007). After updating *m*_*θ*_, the posterior covariance *S*_*θ*_ is set to the negative inverse curvature of $L(\theta ,m_{\lambda })$, evaluated at the new point $\theta =m_{\theta }$ (this is the value of *S*_*θ*_ that minimises the variational energy (Friston et al., 2007)).

Equivalent expressions and updates are implemented for the hyperparameters, with the difference that the temporal regularisation parameter is not adaptive but is fixed at *v*=8. Overall, optimisation proceeds by iteratively updating the approximate posteriors for the parameters and hyperparameters until the change in free energy is less than 0.01. Again, these parameters have been found to provide robust optimisation over a wide variety of problem domains, from EEG source reconstruction to DCM parameter estimation.

## Appendix D Model evidence

The Negative Variational Free Energy is defined as

(29) $$F(m)=\iint q(\theta |y,m)q(\lambda |y,m)\log\left[\frac{p(y,\theta ,\lambda |m)}{q(\theta |y,m)q(\lambda |y,m)}\right]d\theta d\lambda$$

where

(30) p(y,θ,λ|m)=p(y|θ,λ,m)p(θ|m)p(λ|m)

This quantity provides a lower bound on the log model evidence (Beal, 2003). As shown in Wipf and Nagarajan (2009), Penny (2012) (and Eq. (21) in (Friston et al., 2007)) the VL approximation to *F*(*m*) is given by

(31) $$F_{L}(m)=-\frac{1}{2}{\overset{¯}{e}}_{y}^{T}C_{y}^{-1}{\overset{¯}{e}}_{y}-\frac{1}{2}\log|C_{y}|-\frac{N}{2}\log2\pi \;\;-\frac{1}{2}{\overset{¯}{e}}_{\theta }^{T}C_{\theta }^{-1}{\overset{¯}{e}}_{\theta }-\frac{1}{2}\log|C_{\theta }|+\frac{1}{2}\log|S_{\theta }|\;\;-\frac{1}{2}{\overset{¯}{e}}_{\lambda }^{T}C_{\lambda }^{-1}{\overset{¯}{e}}_{\lambda }-\frac{1}{2}\log|C_{\lambda }|+\frac{1}{2}\log|S_{\lambda }|$$

where *N* is the number of data points and the error terms are

(32) $${\overset{¯}{e}}_{y}=y-f(m_{\theta },m)\;{\overset{¯}{e}}_{\theta }=m_{\theta }-\mu_{\theta }\;{\overset{¯}{e}}_{\lambda }=m_{\lambda }-\mu_{\lambda }$$

Generically, factorised variational approximations provide a lower bound on the log model evidence (Beal, 2003). The difference between the true log model evidence and *F*(*m*) is given by the Kullback-Liebler divergence between the true and variational posterior. Thus, as this KL divergence increases the bound becomes less tight and *F*(*m*) will not provide an accurate approximation. It turns out, however, that *F*_*L*_ provides an *approximation* to the model evidence rather than a lower bound (Wipf and Nagarajan, 2009, Penny, 2012) (it can be lower or higher than *F*(*m*)). Empirically, however, it has been shown to provide a better model selection measure than does AIC or BIC (Penny, 2012). The quantity *F*_*L*_(*m*) is the VL model evidence approximation referred to in the paper.

## Appendix E Supplementary data

Application 1

## Footnotes

Appendix E Supplementary data associated with this article can be found in the online version at doi:10.1016/j.neuroimage.2016.12.017.

## References

[bib1] Arand C., Scheller E., Seeber B., Timmer J., Klöppel S., Schelter B. (2015). Assessing parameter identifiability for dynamic causal modeling of fMRI data. Front. Neurosci..

[bib2] Ashburner J., Friston K.J. (2005). Unified segmentation. NeuroImage.

[bib3] Ashburner J., Ridgway G.R. (2013). Symmetric diffeomorphic modeling of longitudinal structural MRI. Front. Neurosci..

[bib4] Ashburner J. (2007). A fast diffeomorphic image registration algorithm. NeuroImage.

[bib5] Aubert-Broche B., Fonov V.S., García-Lorenzo D., Mouiha A., Guizard N., Coupé P., Eskildsen S.F., Collins D.L. (2013). A new method for structural volume analysis of longitudinal brain MRI data and its application in studying the growth trajectories of anatomical brain structures in childhood. NeuroImage.

[bib6] Ballatore C., Lee V.M.-Y., Trojanowski J.Q. (2007). Tau-mediated neurodegeneration in alzheimer's disease and related disorders. Nat. Rev. Neurosci..

[bib7] Beal, M., 2003. Variational Algorithms for Approximate Bayesian Inference. (Ph.D. thesis), Gatsby Computational Neuroscience Unit. University College London.

[bib8] Bernal-Rusiel J.L., Greve D.N., Reuter M., Fischl B., Sabuncu M.R. (2012). For the alzheimer's disease neuroimaging initiative, statistical analysis of longitudinal neuroimage data with linear mixed effects models. NeuroImage.

[bib9] Bishop C. (2006). Pattern Recognition and Machine Learning.

[bib10] Blakemore S.-J., Burnett S., Dahl R.E. (2010). The role of puberty in the developing adolescent brain. Hum. Brain Mapp..

[bib11] Brouwer R.M., Koenis M.M.G., Schnack H.G., van Baal G.C., van Soelen I.L.C., Boomsma D.I., Pol H.E.H. (2015). Longitudinal development of hormone levels and grey matter density in 9 and 12-year-old twins. Behav. Genet..

[bib12] Chen R., Resnick S., Davatzikos C., Herskovits H. (2012). Dynamic bayesian network modelling for longitudinal brain morphometry. Neuroimage.

[bib13] Chumbley J.R., Friston K.J., Fearn T., Kiebel S.J. (2007). A metropolis-hastings algorithm for dynamic causal models. NeuroImage.

[bib14] Daunizeau J., Kiebel S.J., Friston K.J. (2009). Dynamic causal modelling of distributed electromagnetic responses. NeuroImage.

[bib15] Dayan P., Abbott L.F. (2001). Theoretical Neuroscience: Computational and Mathematical Modeling of Neural Systems.

[bib16] Deco G., Jirsa V., Robinson P., Breakspear M., Friston K. (2008). The dynamic brain: from spiking neurons to neural masses and cortical fields. PLoS Comput. Biol..

[bib17] Ducharme S., Albaugh M.D., Nguyen T.-V., Hudziak J.J., Mateos-Pérez J.M., Labbe A., Evans A.C., Karama S., Group B.D.C. (2016). Trajectories of cortical thickness maturation in normal brain development-The importance of quality control procedures. NeuroImage.

[bib18] Erus G., Battapady H., Satterthwaite T.D., Hakonarson H., Gur R.E., Davatzikos C., Gur R.C. (2015). Imaging patterns of brain development and their relationship to cognition. Cereb. Cortex.

[bib19] Evans A., Group B. (2006). The nih mri study of normal brain development. NeuroImage.

[bib20] Fonteijn H.M., Modat M., Clarkson M.J., Barnes J., Lehmann M., Hobbs N.Z., Scahill R.I., Tabrizi S.J., Ourselin S., Fox N.C., Alexander D.C. (2012). An event-based model for disease progression and its application in familial alzheimer's disease and Huntington's disease. NeuroImage.

[bib21] Friston, K.J., Litvak, V., Oswal, A., Razi, A., Stephan, K.E., van Wijk, B.C.M., et al., Bayesian model reduction and empirical Bayes for group (DCM) studies. NeuroImage 128, 2016, 413–431, http://doi.org/10.1016/j.neuroimage.2015.11.015 10.1016/j.neuroimage.2015.11.015 PMC4767224 26569570

[bib22] Friston K.J., Harrison L., Penny W. (2003). Dynamic causal modelling. NeuroImage.

[bib23] Friston K., Mattout J., Trujillo-Barreto N., Ashburner J., Penny W. (2007). Variational free energy and the Laplace approximation. Neuroimage.

[bib24] Friston, K., Ashburner, J., Kiebel, S., Nichols, T., Penny, W., (Eds.). 2007. Statistical Parametric Mapping: The Analysis of Functional Brain Images, Academic Press.

[bib25] Friston K. (2002). Bayesian estimation of dynamical systems: an application to fMRI. NeuroImage.

[bib26] Giedd J.N., Blumenthal J., Jeffries N.O., Castellanos F.X., Liu H., Zijdenbos A., Paus T., Evans A.C., Rapoport J.L. (1999). Brain development during childhood and adolescence: a longitudinal MRI study. Nat. Neurosci..

[bib27] Giedd J.N., Clasen L.S., Lenroot R., Greenstein D., Wallace G.L., Ordaz S., Molloy E.A., Blumenthal J.D., Tossell J.W., Stayer C., Samango-Sprouse C.A., Shen D., Davatzikos C., Merke D., Chrousos G.P. (2006). Puberty-related influences on brain development. Mol. Cell. Endocrinol..

[bib28] Goddings A.-L., Mills K.L., Clasen L.S., Giedd J.N., Viner R.M., Blakemore S.-J. (2014). The influence of puberty on subcortical brain development. NeuroImage.

[bib29] Grimbert F., Faugeras O. (2006). Bifurcation analysis of Jansen's neural mass model. Neural Comput..

[bib30] Group B.D.C. (2011). Total and regional brain volumes in a population-based normative sample from 4 to 18 years: the NIH MRI study of normal brain development. Cereb. Cortex.

[bib31] Hammers A., Allom R., Koepp M., Free S., Myers R., Lemieux L., Mitchell T., Brooks D., Duncan J. (2003). Three-dimensional maximum probability atlas of the human brain, with particular reference to the temporal lobe. Hum. Brain Mapp..

[bib32] Herting M.M., Gautam P., Spielberg J.M., Kan E., Dahl R.E., Sowell E.R. (2014). The role of testosterone and estradiol in brain volume changes across adolescence: a longitudinal structural MRI study. Hum. Brain Mapp..

[bib33] Ingalls B. (2013). Mathematical Modeling in Systems Biology: An Introduction.

[bib34] Lefèvre J., Germanaud D., Dubois J., Rousseau F., de Macedo Santos I., Angleys H., Mangin J.-F., Hüppi P.S., Girard N., Guio F.De. (2015). Are developmental trajectories of cortical folding comparable between cross-sectional datasets of fetuses and preterm newborns?. Cereb. Cortex.

[bib35] Lenroot R.K., Gogtay N., Greenstein D.K., Wells E.M., Wallace G.L., Clasen L.S., Blumenthal J.D., Lerch J., Zijdenbos A.P., Evans A.C., Thompson P.M., Giedd J.N. (2007). Sexual dimorphism of brain developmental trajectories during childhood and adolescence. NeuroImage.

[bib36] Mechelli A., Friston K.J., Frackowiak R.S., Price C.J. (2005). Structural covariance in the human cortex. J. Neurosci.: Off. J. Soc. Neurosci..

[bib37] Mills K.L., Tamnes C.K. (2014). Methods and considerations for longitudinal structural brain imaging analysis across development. Dev. Cogn. Neurosci..

[bib38] Mills K.L., Goddings A.-L., Herting M.M., Meuwese R., Blakemore S.-J., Crone E.A., Dahl R.E., Güroğlu B., Raznahan A., Sowell E.R., Tamnes C.K. (2016). Structural brain development between childhood and adulthood: convergence across four longitudinal samples. NeuroImage.

[bib39] Montague P.R., Dolan R.J., Friston K.J., Dayan P. (2012). Computational psychiatry. Trends Cogn. Sci..

[bib40] Murray J. (2002). Mathematical Biology I: An Introduction.

[bib41] Newman E., Shay C., Hasselmo M. (2012). Malignant synaptic growth and alzheimer's disease. Future Neurol..

[bib42] Oxtoby, N.P., Young, A. L., Fox, N.C., Daga, P., Cash, D.M., Ourselin, S., Schott, J.M., Alexander, D.C., 2014. T.A.D.N. Initiative, Learning Imaging Biomarker Trajectories from Noisy Alzheimer’s Disease Data Using a Bayesian Multilevel Model. In: Bayesian and grAphical Models for Biomedical Imaging, Springer International Publishing, Cham. pp. 85–94.

[bib43] Peelle J.E., Cusack R., Henson R.N.A. (2012). Adjusting for global effects in voxel-based morphometry: gray matter decline in normal aging. NeuroImage.

[bib44] Penny W., Sengupta B. (2016). Annealed importance sampling for neural mass models. PLoS Comput. Biol..

[bib45] Penny W.D. (2012). Comparing dynamic causal models using AIC, BIC and free energy. Neuroimage.

[bib46] Petanjek Z., Judaš M., Šimic G., Rasin M.R., Uylings H.B.M., Rakic P., Kostovic I. (2011). Extraordinary neoteny of synaptic spines in the human prefrontal Cortex. Proc. Natl. Acad. Sci. U. S. Am..

[bib47] Press, W.H., Teukolsky, S.A., Vetterling, W.T., Flannery, B.P., 1992. Numerical Recipes in C (Second Edition), Cambridge, Cambridge.

[bib48] Raznahan A., Shaw P.W., Lalonde F., Stockman M., Wallace G.L., Greenstein D., Clasen L., Gogtay N., Giedd J.N. (2011). How does your cortex grow?. J. Neurosci.: Off. J. Soc. Neurosci..

[bib49] Raznahan A., Lerch J., Lee N., Greenstein D., Wallace G., Stockman M., Clasen L., Shaw P., Giedd J. (2011). Patterns of coordinated anatomical change in human cortical development: a longitudinal neuroimaging study of maturational coupling. Neuron.

[bib50] Sabuncu M.R., Bernal-Rusiel J.L., Reuter M., Greve D.N., Fischl B. (2014). Alzheimer's disease neuroimaging initiative, event time analysis of longitudinal neuroimage data. NeuroImage.

[bib51] Sengupta B., Friston K.J., Penny W.D. (2016). Gradient-based MCMC samplers for dynamic causal modelling. NeuroImage.

[bib52] Shaw P.W., Kabani N.J., Lerch J.P., Eckstrand K., Lenroot R., Gogtay N., Greenstein D., Clasen L., Evans A.C., Rapoport J.L., Giedd J.N., Wise S.P. (2008). Neurodevelopmental trajectories of the human cerebral cortex. J. Neurosci.: Off. J. Soc. Neurosci..

[bib53] Smith S.M., Fox P.T., Miller K.L., Glahn D.C., Fox P.M., Mackay C.E., Filippini N., Watkins K.E., Toro R., Laird A.R., Beckmann C.F. (2009). Correspondence of the brain's functional architecture during activation and rest. Proc. Natl. Acad. Sci. USA.

[bib54] Stephan K.E., Tittgemeyer M., Knösche T.R., Moran R.J., Friston K.J. (2009). Tractography-based priors for dynamic causal models. NeuroImage.

[bib55] Stephan K.E., Iglesias S., Heinzle J., Diaconescu A.O. (2015). Translational perspectives for computational neuroimaging. Neuron.

[bib56] Taki Y., Hashizume H., Sassa Y., Takeuchi H., Asano M., Asano K., Kotozaki Y., Nouchi R., Wu K., Fukuda H., Kawashima R. (2012). Correlation among body height, intelligence, and brain gray matter volume in healthy children. NeuroImage.

[bib57] Tallinen T., Chung J.Y., Rousseau F., Girard N., Lefèvre J. (2016). On the growth and form of cortical convolutions. Nat. Phys..

[bib58] Tamnes C.K., Walhovd K.B., Dale A.M., Ostby Y., Grydeland H., Richardson G., Westlye L.T., Roddey J.C., Hagler D.J., Due-Tønnessen P., Holland D., Fjell A.M. (2013). A.D.N. initiative, brain development and aging: overlapping and unique patterns of change. NeuroImage.

[bib59] Tau, G., Peterson, B., Normal development of brain circuits, Neuropsychopharmacology, 2009. 10.1038/npp.2009.115 PMC3055433 19794405

[bib60] Taylor S.J., Whincup P.H., Hindmarsh P.C., Lampe F., Odoki K., Cook D.G. (2001). Performance of a new pubertal self-assessment questionnaire: a preliminary study. Paediatr. Perinat. Epidemiol..

[bib61] Thompson, D., 1945. On Growth and Form, Cambridge.

[bib62] Valdes-Sosa P.A., Roebroeck A., Daunizeau J., Friston K. (2011). Effective connectivity: influence, causality and biophysical modeling. NeuroImage.

[bib63] Warren J.D., Rohrer J.D., Schott J.M., Fox N.C., Hardy J., Rossor M.N. (2013). Molecular nexopathies: a new paradigm of neurodegenerative disease. Trends Neurosci..

[bib64] Weiskopf N., Mohammadi S., Lutti A., Callaghan M.F. (2015). Advances in MRI-based computational neuroanatomy: from morphometry to in-vivo histology. Curr. Opin. Neurol..

[bib65] Wierenga L.M., Langen M., Oranje B., Durston S. (2014). Unique developmental trajectories of cortical thickness and surface area. NeuroImage.

[bib66] Wilson H. (1999). Spikes, Decisions and Actions: The Dynamical Foundations of Neuroscience.

[bib67] Wipf D., Nagarajan S. (2009). A unified bayesian framework for MEG/EEG source imaging. Neuroimage.

[bib68] Young A.L., Oxtoby N.P., Daga P., Cash D.M., Fox N.C., Ourselin S., Schott J.M., Alexander D.C. (2014). Alzheimer's disease neuroimaging initiative, a data-driven model of biomarker changes in sporadic alzheimer's disease. Brain: J. Neurol..

[bib69] Zeidman P., Maguire E.A. (2016). Anterior hippocampus: the anatomy of perception, imagination and episodic memory. Nat. Rev. Neurosci..

[bib70] Ziegler G., Penny W.D., Ridgway G.R., Ourselin S., Friston K.J. (2015). A.D.N. initiative, estimating anatomical trajectories with Bayesian mixed-effects modeling. NeuroImage.

