# Supplementary material for: Multivariate dynamical modelling of structural change during development
Source: Neuroimage. 2017 Feb 15;147:746–62. doi: 10.1016/j.neuroimage.2016.12.017 (PMC5315058; doi:10.1016/j.neuroimage.2016.12.017)
Supplement: Application 1 [file mmc1.pdf]

Supplementary material

brain region	lds - girls	lds - boys	poly - girls	poly - boys
Ins	8.7	9.4	9.2	9.4
AntCingG	8.6	9.3	9.9	9.4
FroG	8.6	9.3	9.2	9.4
InfFroG	8.4	9.1	9.2	9.0
MidFroG	8.4	9.2	9.1	9.5
SupFroG	8.5	9.3	9.0	9.9
PrcG	8.2	8.6	6.2	8.3
RecG	8.2	6.1	6.1	7.8
AntMedTemL	8.6	9.9	6.4	11.2
AntLatTemL	8.7	9.6	9.6	10.5
SupTemG	8.2	8.7	8.5	6.6
InfMidTemG	8.6	9.1	6.6	9.1
FusG	8.3	9.0	8.0	7.6
PosTemL	8.1	8.7	7.0	8.0
PosCinG	7.6	6.1	6.1	6.8
PoCG	6.1	6.1	6.1	6.1
LatParL	7.6	6.1	7.0	6.5
SupParG	6.1	6.1	6.1	6.7
Cun	6.1	6.1	6.1	6.1
LatOccL	6.1	6.1	6.1	6.4
LinG	6.1	6.1	6.1	6.1
Hipp	16.1	16.5	16.5	13.9
Amy	15.1	16.1	22.0	14.8
Put	8.0	8.7	7.4	6.7
CauNuc	8.2	8.9	9.3	8.2
Tha	8.0	6.1	6.1	6.1

Table 1: Numerically estimated age in years of peak volume (given the age range of the included subjects) in 26 brain regions using trajectories from multivariate linear dynamical system (lds) and cubic polynomials (poly) from girls and boys respectively. Dynamical system model refers to the highest evidence model with gender-specific self-connections, puberty and alpha growth dynamics. Cubic polynomial model refers to having independent coefficients across gender and all brain regions.
